# A 4-factor perspective of the patient-practitioner orientation scale (PPOS): a deeper understanding of patient-centredness

**DOI:** 10.1186/s12909-022-03867-w

**Published:** 2022-11-29

**Authors:** Yinan Jiang, Jing Wei, Lili Shi, Jinya Cao, Boheng Zhu, Xia Hong

**Affiliations:** Department of Psychological Medicine, Peking Union Medical College Hospital, Chinese Academy of Medical Sciences & Peking Union Medical College, Shuaifuyuan1, Dongcheng District, Beijing, 100730 People’s Republic of China

**Keywords:** Doctor–patient relationship, Patient-centredness, Patient-practitioner orientation scale, Reliability, Validity

## Abstract

**Background:**

Although patient-centred medical services are widely recognized and accepted, how to define and evaluate them remains a controversial topic.

**Objectives:**

This study attempts to evaluate the underlying structure of the Patient-Practitioner Orientation Scale (PPOS) with a homogenous population and clarify the connotation of patient-centredness.

**Methods:**

In this cross-sectional study, 279 7th year Chinese medical students in were selected to examine the internal structure of the PPOS by means of internal consistency, exploratory, and confirmatory factor analyses.

**Results:**

Both the two-factor model and the four-factor model showed acceptable internal consistency and structural validity. The four-factor model that endorsed the implicit attitude towards the doctor–patient relationship outperformed the two-factor model in terms of adaptability.

**Conclusions:**

The PPOS has good psychometric attributes, as evaluated by Chinese medical students. This article attempts to explore patient-centredness from the perspective of implicit attitudes that affect the doctor–patient relationship and resummarizes the four factors. These four dimensions may suggest a deeper attitude towards the doctor–patient relationship, while “sharing information” or “caring about” the “patient” is the behaviour and preference expressed on the basis of these four attitudes, which is the result rather than the cause.

**Practice implications:**

Understanding the underlying attitudes towards the doctor–patient relationship can help to construct a patient-centred medical service concept and improve the doctor–patient relationship in medical education courses and the system design of medical activities.

**Supplementary Information:**

The online version contains supplementary material available at 10.1186/s12909-022-03867-w.

## Introduction

Patient-centred medical care has been valued and researched worldwide [1–5]. Patient-centred care is defined as medical care that respects and conforms to the patient’s personal preferences, needs and values and ensures that clinical decisions are aligned with the patient’s values [6, 7]. A study confirmed that patient-centred care facilitated more positive medical outcomes, including improved patient satisfaction and adherence to treatment, reduced malpractice and claims, improved doctor–patient communication, improved professional satisfaction for doctors, reduced consultation time during medical activities and reduced medical insurance costs [8–13].

To improve the level of patient-centred services, researchers are developing tools to evaluate patient-centred professional attitudes more accurately [12, 14–18]. Among them, the Patient-Practitioner Orientation Scale (PPOS)^1^ is a tool that was developed by the American scholar [15] and is used to evaluate the attitudes of doctors, medical students and patients towards patient centredness. This tool has been widely used and translated into different languages, and its reliability and validity have been tested in several countries [19–26]. By assessing the degree of identification of subjects with certain behaviours and attitudes in the interaction between doctors and patients, PPOS reflects their attitude towards “patient-centred” care, and through model analysis, two dimensions are further formed: one is the attitude towards holistic medicine or biomedical medicine (reflected by the caring subscale); the second is doctors’willingness to share decision-making power (reflected by the sharing subscale). It must be acknowledged that, in theory, the use of these two dimensions to divide patient-centred attitudes is understandable. However, in practice, both doctors’ and patients’ attitudes towards patient-centred care are not directly reflected in these two dimensions. We have found from the actual observation of teaching that understanding the concept of patient-centredness and recognizing the attitude of patient-centredness is not the same as the patient-centred doctor–patient practice in actual clinical work. From this, we speculate that behind patient-centred behaviour, there are deeper factors that affect the behaviour of doctors. Furthermore, there was not intended to be a dichotomy of being either doctor/disease-centred or patient-centred. The factors that influence one’s explicit attitude and behaviour may provide a deeper understanding of related issues. Previous studies on the reliability and validity of the PPOS also show that the 2-dimensional model seems to be unstable. For example, in a 2012 study by Pereira et al. [25], the overall Cronbach’s α value of the Portuguese version of the PPOS questionnaire was 0.605 in Brazilian residents, medical students, and patients. Confirmatory factor analysis results suggested that the two-factor model is acceptable. However, a study conducted in Spain in 2020 indicated that the 2-factor model only obtained a good fit to the data after excluding 8 items; therefore, there is perhaps the possibility of a 3-factor model for PPO S[26].

Since 2016, Chinese scholars have also begun to use the PPOS to explore the attitudes of Chinese medical staff, medical students and patients towards patient-centred medical care [27–30]. In 2017, Wang et al. [30] took 187 clinicians and 831 patients as research participants to study the reliability and validity of the Chinese version of the PPOS and modified the tool to form the revised Chinese version of the PPOS questionnaire. The results showed that the 18-item Chinese PPOS is not ideal in terms of reliability and validity. Therefore, the revised Chinese version of the PPOS (CR-PPOS) was formed, with the total number of items reduced to 11 and the original 2 dimensions remaining, including 6 items of the sharing dimension and 5 items of the care dimension. The CR-PPOS obtained better psychometric indicators.

In addition to the differences presented by the above PPOS structure studies, in 2005, Epstein et al. also proposed **a** 4-factor model, which includes patient perspective, social background, shared understanding and shared power, to promote patient-centred care [31]. Consequently, the connotation of the patient-centred concept remains to be further explored. Structural analysis of PPOS questionnaires with more homogenous research objects may help to better reveal this problem. At Peking Union Medical College & Chinese Academy of Medical Sciences, there is a long history of education pertaining to doctor–patient relationships and clinical communication to encourage medical students to develop patient-centred communication skills. We took 7th year medical students majoring in clinical medicine at Peking Union Medical College (equivalent to 3rd year medical students in the USA) who had received training in doctor–patient relationships and clinical communication skills in their compulsory courses as the research object to evaluate the structural validity and internal consistency of the 18 items of the Chinese PPOS and to attempt to explore the connotation of the concept of patient-centredness.

## Methods

### Description of the instrument

The Patient-Practitioner Orientation Scale (PPOS) [15] is a scale that assesses the attitudes of physicians, medical students, patients, and the general population towards the doctor–patient relationship. The scale contains 18 items, including two subscales related to medical interviews: sharing and care [22]. The two subscales each contain 9 items. The sharing subscale mainly assesses whether respondents believe that doctors and patients should have equal power and control and to what extent doctors should share information with patients. The caring subscale assesses whether respondents believe that the patients’ expectations, feelings, and preferences are key factors in the doctor–patient relationship. The PPOS scale uses a 6-point Likert scale (from strongly agree to strongly disagree). Higher total PPOS and subscale scores indicate that respondents preferred a patient-centred approach. In contrast, the lower the score of the sharing subscale is, the more inclined the respondents are to a physician-centred approach; the lower the score on the care subscale is, the greater the preference for a disease-centred approach.

### Study design and participants

The study was conducted between September 2010 and October 2014. We conducted a cross-sectional survey of 279 medical students from Peking Union Medical College after obtaining the permission of the original author of PPOS and the approval of the Ethics Review Committee of Peking Union Medical College. Peking Union Medical College, which was founded in 1917, has been under a unified management system with Peking Union Medical College Hospital since 1957. It is the top medical research and education institution in China. The clinical medicine program of Peking Union Medical College is an 8-year scheme, including premedical training (2 and a half years), basic medicine (1 and a half years), clinical medicine (3 years), and research training (1 year).

Seventh year medical students were selected as the research samples because, on the one hand, they have completed the study of basic medicine, some clinical medicine, and have gained medical clinical communication skills and certain medical knowledge; on the other hand, they have not independently undertaken work as doctors. Therefore, both physician and nonphysician perspectives are taken into account. From both patient and physician perspectives, the quality of research on this group and the rate of questionnaire completion are high; therefore, this study can provide a beneficial supplement for the application of PPOS and patient-centred understanding. The assessments were anonymous and voluntary and did not affect the subjects’ grades or degrees.

Since the minimum sample size for exploratory factor analysis (EFA) is 5–10 times the number of tool items, the minimum sample size for confirmatory factor analysis (CFA) is 10–20 times the number of tool items [32]. Therefore, the current sample size meets the needs of the study.

### Translation and cultural adaptation

In late 2003, the Chinese Medical Doctor Association invited scholars with academic backgrounds in medicine, public health and communication, as well as Chinese and English scholars, to translate the English PPOS into Chinese (Putonghua) after obtaining permission from the original author for Chinese translation and development. The bilingual PPOS version was then sent separately to five other senior health practitioners for further advice and modifications. The Chinese version of the PPOS (C-PPOS) was then back-translated into English to verify its accuracy. From 2007 to 2009, we used the questionnaire among medical students at Peking Union Medical College & Chinese Academy of Medical Sciences, and students reported that the questionnaire was well understood and culturally appropriate.

### Psychometric properties assessment

In this study, the reliability, validity and discriminative ability of the C-PPOS were tested to evaluate their psychometric properties, and corresponding revisions were made to the C-PPOS on this basis. In terms of reliability evaluation, internal consistency was used. Exploratory factor analysis (EFA) and confirmatory factor analysis (CFA) were used to assess structural validity [32]. Content validity was evaluated by examining the correlation between the score of each item and the score of the subscale to which the item belongs. We assessed discriminant validity by comparing the threshold (CR) for each item.

### Statistical analysis

We used EpiData V.3.1 software to build the database. IBM SPSS V.22.0 and AMOS V.22.0 were used for data analysis. The maximum likelihood estimation is used for missing values. Cronbach’s α was used to evaluate the internal consistency of the PPOS and subscales. In general, a Cronbach’s α value not less than 0.6 is considered acceptable for tools with relatively few items (i.e., no more than 6 items). For C-PPOS, in its entirety, a Cronbach’s α value not less than 0.7 is considered acceptable [33].

For exploratory factor analysis, we used principal component analysis and the Kaiser normalized maximum variance method to analyse the data. KMO > 0.8 was considered suitable for factor analysis. The eigenvalues (greater than 1) of the subscale (ROUND 1 of EFA) were extracted. In EFA Round 1, the following criteria were established for entries to be retained: (1) they had to have factor loads greater than 0.4 in either dimension, (2) the factor load could not exceed 0.4 in any two dimensions at the same time, and (3) there had to be at least three last reserved items on each dimension [34].

For confirmatory factor analysis, we used maximum likelihood analysis. The main adjustment indicators of the validation model include the normed-fit index (NFI), incremental fit index (IFI), comparative fit index (CFI),goodness-of-fit index (GFI), adjusted goodness-of-fit index (AGFI), root mean square error of approximation (RMSEA), and χ2/df. GFI, AGFI, NFI, IFI and CFI > 0.9 suggested an ideal model fit and an RMSEA value < 0.08, and the recommended χ2/df ratio was 1 to 3[35].

The correlation between each item and each factor was expressed by the Pearson correlation coefficient. The tool’s discrimination ability was evaluated by dividing respondents with the highest (top 27%) and lowest (bottom 27%) scores into two groups and comparing their CR values for each item [36].

## Results

### Participants

A total of 279 questionnaires were distributed and 242 were returned, of which 235 were valid. Among the students who completed the assessment, 146 (60.3%) were female, aged 24.45 ± 0.942 years, 182 (77.45%) came from urban areas, and 212 (90.2%) were of Han nationality (China’ s main nationality, distributed all over the country), all of whom were unmarried.

### Validity

#### Construct validity

##### Exploratory factor analysis of C-PPOS

In exploratory factor analysis (EFA), the Kaiser–Meyer–Olkin sampling suitability quantile was 0.829 (*P* < 0.001). Exploratory factor analysis extracted four factors with principal component eigenvalues greater than 1, explaining 48.678% of the total variance. The four factors could explain 18.427, 10.537, 10.453 and 9.261% of the total variance. The factor loading of each item was > 0.4, and there were 3 items with the fewest items among the 4 factors.

##### Confirmatory factor analysis of C-PPOS

Two-factor models and four-factor models were used for confirmatory factor analysis. According to the feedback of the confirmatory factor analysis of the 4-factor model and the practical significance analysis, each factor item was adjusted to obtain the revised 4-factor model. The CFA results of the revised 4-factor model showed a significant improvement in model fitting compared with the 2-factor model (Table 1). For the specific content of the 2-factor model and the modified 4-factor model, please refer to Attachments 1 and 2.

**Table 1:** Model fit indices for 2-factor and 4-factor CFA models of the PPOS

Model fit indicator	2-Factor		4-factor			
**χ**^2^/df	2.13		1.90			
*p* Value	0.000		0.000			
RMSEA	0.07		0.06			
GFI	0.87		0.90			
AGFI	0.83		0.86			
NFI	0.69		0.73			
IFI	0.81		0.85			
CFI	0.80		0.85			
Factors	Care	Sharing	Factor 1	Factor 2	Factor 3	Factor 4
Internal Consistency Cronbach’s α	.574	.685	.470	.547	.770	.554

*AGFI* adjusted goodness-of-fit index, *CFA* confirmatory factor analysis, *CFI* comparative fit index, *C-PPOS* Chinese Patient–Practitioner Orientation Scale, *CR-PPOS* Chinese-revised Patient–Practitioner Orientation Scale, *GFI* goodness-of-fit index, *IFI* incremental fit index, *NFI* normed-fit index, *RMSEA* root mean square error of approximation

Factor 1: Whether medicine is considered omnipotent

This set of questions all reflects the level of belief in biomedical technology

Factor 2: Whether the patients are recognized as competent

This set of questions all reflects attitudes towards the patient’s ability to play an active role in treatment

Factor 3: How to view doctors’ interpersonal style

This set of questions all reflects the cognitive attitude of doctors in addition to medical treatment, such as the doctor’s personality traits and communication style

Factor 4: Whether recognition of patients’ feelings affects patients’ medical behaviour and outcome

This group of questions reflects the importance of the patient’s feelings during the treatment process

#### Content validity

In terms of content validity, the correlation and significance between the scores of each item in the revised 4-factor model and the scores of the corresponding subscales are also significantly improved compared with the 2-factor model. For detailed results, please refer to Table 2.

**Table 2:** Correlation and validity of the PPOS

	Discriminant validity^a^	Content validity^b^					
		2-Factor		4-Factor			
		Care	Sharing	Factor 1	Factor 2	Factor 3	Factor 4
Item 1	.000		0.605^***^	0.665^**^			
Item 2	.000	0.489^**^		0.526^**^			
Item 3	.000	0.320^**^		0.325^**^			
Item 4	.000		0.673^***^		0.735^***^		
Item 5	.000		0.704^***^			0.696^***^	
Item 6	.000	0.538^**^				0.551^***^	
Item 7	.000	0.690^**^				0.672^***^	
Item 8	.000		0.362^***^		0.492^***^		
Item 9	.051		−0.256^**^				0.223^**^
Item 10	.018		−0.002				0.473^***^
Item 11	.000	0.580^**^				0.564^***^	
Item 12	.000		0.365^***^		0.365^***^		
Item 13	.001	0.124		0.167^*^			
Item 14	.000	0.536^**^				0.539^***^	
Item 15	.000		0.312^**^				0.623^***^
Item 16	.000	0.560^**^				0.556^*^	
Item 17	.000	0.181^*^					0.543^*^
Item 18	.000		0.306^*^		0.346^*^		

^a^Values in boxes indicate *p* values for each item

^b^values in boxes indicate factor loadings for each item; ***: *p* < 0.001; **: *p* < 0.05; *: without *p* values; without an asterisk: *p* ≥ 0.05

Factor 1: Whether medicine is considered omnipotent

Factor 2: Whether the patients are recognized as competent

Factor 3: How to view doctors’ interpersonal style

Factor 4: Whether recognition of patients’ feelings affects patients’ medical behaviour and outcome

#### Discriminant validity

Except for Item 9, the *p*-values of CRs in all items were less than 0.05, reaching a statistically significant level. Item 9 has a *p*-value of 0.051. (Table 2).

## Discussion

In this study, the results indicate that the 18-item Chinese PPOS has two different perspectives with medical students as research participants, which were the traditional 2-factor model (Care and Sharing) and the exploratory 4-factor model (medical technology comprehensiveness; whether the patient is competent; doctor’s interpersonal style; and whether the patient’s feelings matter).

First, for the traditional 2-factor model, we re-evaluated the reliability and validity of the tool using the internal consistency index and confirmatory factor analysis. The overall Cronbach’s α value of the questionnaire was 0.763, the care subscale score was 0.574, and the sharing subscale score was 0.685.Confirmatory factor analysis results support an adequate model fit (χ^2^/df = 2.13, *p*<0.001, RMSEA = 0.07, GFI = 0.87, AGFI = 0.83, NFI = 0.69, IFI = 0.81, and CFI = 0.80). Compared with the Chinese version of the PPOS study in 2017, the above results show significant improvement in both reliability and validity.^14^ In the 2017 study, the overall Cronbach’s α value of the questionnaire was 0.668, the care subscale score was 0.493, and the sharing subscale score was 0.575.Confirmatory factor analysis results suggested an acceptable model fit (χ^2^/df = 5.04，*p*<0.001，RMSEA = 0.11, GFI = 0.76, AGFI = 0.70, NFI = 0.52, IFI = 0.58, and CFI = 0.57), which is comparable to the results of the Portuguese version of the PPOS [25]. Therefore, the Chinese version of the PPOS of 18 items is also applicable to the Chinese population.

Second, through exploratory factor analysis and confirmatory factor analysis, this study found that the 18-item PPOS scale could extract 4 factors with principal component eigenvalues greater than 1.The 4-factor model also has good reliability and validity performance. The Cronbach’s α value of Factor 1 was 0.470, Factor 2 was 0.547, Factor 3 was 0.770, and Factor 4 was 0.554. The results of the confirmatory factor analysis showed that χ^2^/df = 1.89, *p*<0.001, RMSEA = 0.06, GFI = 0.90, AGFI = 0.87, NFI = 0.73, IFI = 0.85, and CFI = 0.85.This result is statistically superior to the 2-factor model, but the practical value of the 4-factor model depends on its ability to better explain the doctor–patient relationship.

By analysing the meaning of items, this study leads to the conclusion that the 4-factor model evaluates the attitudes of interviewees from the following perspectives: ① Factor 1 evaluated whether medicine was considered omnipotent;② Factor 2 evaluated whether the patients were recognized as competent;③ Factor 3 evaluated how to view doctors’ interpersonal style; and ④ Factor 4 evaluated whether recognition of patients’ feelings would affect patients’ medical behaviour and outcome.

Factor 1 includes the following items:

**Table:** 

1. The doctor is the one who should decide what is talked about during a visit. 2. Although health care is less personal these days, it is a small price to pay for medical advances. 3. The most important part of the standard medical visit is the physical exam. 13. A treatment plan cannot succeed if it conflicts with a patient’s lifestyle or values.	

According to our analysis, Factor 1 is related to whether the participants agree that “medicine is an all-powerful science”. In theory, the more that participants agreed that medicine was “all-powerful,” the more physician-centred they tended to be (Item 1),the more likely they were to consider that the physical examination was the most important (Item 3), and the more likely they were to suppress their inner feelings (Items 2 and 13). Since this study used Chinese medical students as the research participants, we speculated that the aforementioned outcomes might be related to the fact that medicine is often considered omnipotent in traditional Chinese culture, and it is considered that doctors have the capacity to accurately predict and judge patients’ conditions. For example, the story by Han Fei of the miraculous doctor Bian Que. meeting Duke Cai Huan describes the doctor discovering the duke’s potential disease through observation and predicting its occurrence and development. On the one hand, such stories increase the public’s trust and respect for doctors, but on the other hand, they may also raise the people’s psychological expectations for doctors to possess “perfect medical skills”. Therefore, the more the patient believes in the existence of such a doctor whose medical knowledge is sufficient to guide all of the patient’s treatment, the more likely he or she is to have a doctor/disease-centred attitude. In contrast, the more the imperfection and uncertainty of medicine itself is acknowledged, the less likely it is for the patient to have a doctor/disease-centred attitude.

Factor 2 includes the following entries:

**Table:** 

4. It is often best for patients if they do not have a full explanation of their medical condition. 8. Many patients continue asking questions even though they are not learning anything new. 12. When patients disagree with their doctor, it is a sign that the doctor does not have the patient’s respect and trust. 18. When patients look up medical information on their own, this usually confuses them more than it helps.	

According to our analysis, Factor 2 reflects whether patients can make desirable choices when faced with medical uncertainties. In the medical decision-making process, because the doctor uses diagnostic technology, aetiology and prognosis, treatment and prevention strategies for the disease are fully understood by the doctor and therefore he or she may question the ability of the patient to make clinical decisions (items 4 and 8, items 12 and 18), thinking that the patient’s positive participation is meaningless. However, greater attention should be given to the fact that the patient is the most familiar with his or her experience of the disease, habits and behaviours, risk attitudes, values and preferences and the social environment, and that these factors may contain important information that can influence the therapeutic effect. Therefore, whether patients are recognized as having the ability to choose in medical decision-making reflects attitudes towards the “doctor/disease centred” or “patient-centred” medical service models. This attitude varies among different populations, according to previous research data [21, 23, 29, 37–46]. In more economically developed regions, both doctors and patients are more likely to develop a patient-centred attitude, while on the other hand, people in less developed areas are more likely to develop a doctor/disease-centred attitude. A possible cause of this difference is that the patients in areas with high levels of regional economic and social development received higher levels of education and developed greater awareness of their needs; thus, in medical treatment, they were better able to express their expectations and desires to receive more attention and were more inclined to adopt a patient-centred attitude towards medical services.

Factor 3 includes the following entries:

**Table:** 

5. Patients should rely on their doctors’ knowledge and not try to diagnose their conditions on their own. 6. When doctors ask many questions about a patient’s background, they are prying too much into personal matters. 7. If doctors are truly good at diagnosis and treatment, the way they relate to patients is not that important. 11. If a doctor’s primary tools are being open and warm, the doctor will not have much success. 14. Most patients want to get in and out of the doctor’s office as quickly as possible. 16. It is not that important to know a patient’s culture and background to treat the person’s illness.	

According to our analysis, Factor 3 is concerned with the expectations of the assessed interpersonal style of doctors. Since the middle of the last century, attention has been given to the influence of doctors’ interpersonal style on doctor–patient relationships [43, 47–50]. In previous studies, doctors’ interpersonal styles were divided into paternalistic and partnership styles. The paternalistic interpersonal style is very common in medical service systems across the world; in this interpersonal style, in a sense, the doctor plays a similar role to a parent, and in this style, the doctor is well intentioned and accepts greater responsibility, which may lead to good health outcomes while simultaneously creating and sustaining an unhealthy dependency. A partnership interpersonal style is one in which people work together towards a common goal. Their relationship is based on mutual respect for each other’s skills and abilities and the advantages of being able to combine those resources to achieve beneficial outcomes. Successful partnerships are equal, with partners making decisions and sharing responsibilities [51]. We believe that each item in Factor 3 reflects the evaluation of doctors’ interpersonal styles. Previous studies have shown that East Asian cultures, such as Japan and South Korea, are more physician- and disease-centred than Europe and North America, which are at the same economic level [37, 41, 43, 45]. This may be related to the fact that traditional medicine in East Asian cultural areas is deeply influenced by Confucianism. The moral code of doctors in the Inner Canon of the Yellow Emperor (the most authoritative text of early medical theory and drug therapy) emphasizes that “doctors should have the same responsibilities as parents”, which means that doctors should treat patients as parents treat children, and doctors should adopt a more paternalistic interpersonal style.

Factor 4 includes the following entries:

**Table:** 

9. Patients should be treated as if they were partners with the doctor, equal in power and status. 10. Patients generally want reassurance rather than information about their health. 15. The patient must always be aware that the doctor is in charge. 17.Humour is a major element of the doctor’s treatment of the patient.	

In our analysis, Factor 4 focuses on whether the participants accept that the patients’ emotions and feelings affect their medical decisions, that is, whether patients make so-called “irrational” choices influenced by their feelings even when they know that there may be a better or worse rational choice. Many previous studies have focused on irrational decision-making; for instance, in behavioural studies based on financial distribution, the vast majority of participants are influenced by emotions, such as sympathy, disappointment and humiliation, to make irrational decisions [52]. Thus, we hypothesized that the more aware the participants were of the influences of emotions and feelings on human behaviour, the more likely they were to be patient-centred in their medical activities. According to our analysis, each item of Factor 4 evaluates whether the participant agrees with the patient’s feeling of being respected (Item 9), whether they are comforted emotionally (Item 10), whether they feel valued by the doctor (Item 15), and whether the doctor is capable of relieving emotional tension (Item 17).

In conclusion,individuals who frequently accept medical limitations are more apt to respect patients’ abilities, tend to adopt a partnership style in doctor–patient communication, understand that people are more likely to be affected by emotions, and are more willing to share power in medical activities, and respect patients’ right to know and agency in decision-making (sharing) in medical activities. They are also more inclined to respect their feelings (care).

The contribution of this paper is reflected in the following aspects. First, there was only one previous PPOS study in China, and the results showed that the application effect of PPOS in China was not satisfactory, and many of the questionnaire items were invalidated. In this paper, the reliability and validity of this tool are retested in the context of China in an attempt to provide new support for the effective use of PPOS in China. Second, this paper discusses patient-centred care from the perspective of medical students rather than the traditional perspective of doctors and patients. Selecting medical students with a certain theoretical basis and clinical practice experience as research objects is a beneficial extension of PPOS-related research and helps to improve patient-centred care from the perspective of medical education. Third, by further exploring the factor structure of PPOS, the original two factors are extended to four factors at the attitude level, which is conducive to a deeper understanding and identification of patient-centredness.

Limitations and Prospects: The advantage of this paper is that through the four-factor model, the implicit attitude behind the behaviour of the doctor–patient relationship is explored from a psychological perspective, which has positive significance for better promoting the establishment of a patient-centred doctor–patient alliance in medical education from a psychological perspective. However, due to data limitations, more data and research are needed to further support the relevant conclusions in the future.

## Conclusions

This paper attempts to use Chinese data to retest PPOS. The results show that the original 2-factor model has good reliability and validity, which shows that the PPOS also has good applicability in China. “Caring” and “Sharing” under the two-factor model examine the aspect of doctor–patient relationship concerning whether doctors are willing to “sharing” or “care”. Further analysis in this study showed that the statistical results of the four-factor model were more significant. This paper attempts to explore patient-centredness from the perspective of implicit attitudes affecting the doctor–patient relationship and resummarizes the four factors. The findings can be summarized as follows: whether medicine was considered omnipotent; whether patients were recognized as competent; how to view doctors’ interpersonal style; and whether recognition of patients’ feelings affects patients’ medical behaviour and outcome. The above four dimensions may suggest a deeper attitude towards the doctor–patient relationship, and “sharing information” or “caring for patients” are the behaviours and preferences that are manifested on the basis of these four attitudes and are the result rather than the cause.

### Practical implications

Our results suggest that understanding the underlying attitudes towards the doctor–patient relationship can help to conceptualize a patient-centred medical service, thereby improving the doctor–patient relationship by implementing it in medical education courses and in the system design of medical activities.

The significance of a 4-factor model of PPOS lies in the fact that if we want to treat patients as the centre of the service, this idea should be taught from the start of medical education and the objective should be accomplished in a more specific dimension through a curriculum designed to strengthen relevant courses. Furthermore, patient-centredness should be taught from the concept level to affect the implicit attitudes of medical students, to guide them in future clinical work with respect to patients and to establish a good cooperative relationship with the patient.

## Supplementary Information

**Additional file 1: Attachment 1**. 2-factor model. **Attachment 2**. 4-factor model.

## Data Availability

The dataset supporting the conclusions of this article is included within the article.

## Footnotes

1 For details on PPOS, see Krupat E, (1999).
