# Supplementary material for: A 4-factor perspective of the patient-practitioner orientation scale (PPOS): a deeper understanding of patient-centredness
Source: BMC Med Educ. 2022 Nov 29;22:818. doi: 10.1186/s12909-022-03867-w (PMC9706840; doi:10.1186/s12909-022-03867-w)
Supplement: Supplementary file 1 — Additional file 1: Attachment 1. 2-factor model. Attachment 2. 4-factor model. [file 12909_2022_3867_MOESM1_ESM.docx]

**Attachment 1.** 2-factor model

| Factor | Items |
| --- | --- |
| Factor 1：Sharing | 1. The doctor is the one who should decide what is talked about during a visit.  4. It is often best for patients if they do not have a full explanation of their medical condition  5. Patients should rely on their doctors’ knowledge and should not try to diagnose their conditions on their own.  8. Many patients continue asking questions even though they are not learning anything new.  9. Patients should be treated as if they were partners with the doctor, equal in power and status.  10. Patients generally want reassurance rather than information about their health.  12. When patients disagree with their doctor, this is a sign that the doctor does not have the patient’s respect and trust.  15. The patient must always be aware that the doctor is in charge.  18. When patients look up medical information on their own, this usually confuses them more than it helps. |
| Factor 2：Care | 2. Although health care is less personal these days, it is a small price to pay for medical advances.  3. The most important part of the standard medical visit is the physical exam.  6. When doctors ask many questions about a patient’s background, they are prying too much into personal matters.  7. If doctors are truly good at diagnosis and treatment, the way they relate to patients is not that important.  11. If a doctor’s primary tools are being open and warm, the doctor will not have much success.  13. A treatment plan cannot succeed if it conflicts with a patient’s lifestyle or values.  14. Most patients want to get in and out of the doctor’s office as quickly as possible.  16. It is not that important to know a patient’s culture and background to treat the person’s illness.  17.Humour is a major element of the doctor’s treatment of the patient. |

**Attachment 2.** 4-factor model

| Factor | Items |
| --- | --- |
| Factor 1：Whether medicine is considered omnipotent | 1. The doctor is the one who should decide what is talked about during a visit.  2. Although health care is less personal these days, it is a small price to pay for medical advances.  3. The most important part of the standard medical visit is the physical exam.  13. A treatment plan cannot succeed if it conflicts with a patient’s lifestyle or values. |
| Factor 2：Whether the patients are recognized as competent | 4. It is often best for patients if they do not have a full explanation of their medical condition  8. Many patients continue asking questions even though they are not learning anything new.  12. When patients disagree with their doctor, this is a sign that the doctor does not have the patient’s respect and trust.  18. When patients look up medical information on their own, this usually confuses more than it helps. |
| Factor 3：How to view doctors' interpersonal style | 5. Patients should rely on their doctors’ knowledge and not try to diagnose their conditions on their own.  6. When doctors ask many questions about a patient’s background, they are prying too much into personal matters.  7. If doctors are truly good at diagnosis and treatment, the way they relate to patients is not that important.  11. If a doctor’s primary tools are being open and warm, the doctor will not have much success.  14. Most patients want to get in and out of the doctor’s office as quickly as possible.  16. It is not that important to know a patient’s culture and background to treat the person’s illness. |
| Factor 4：Whether recognition of patients' feelings affects patients' medical behaviour and outcome | 9. Patients should be treated as if they were partners with the doctor, equal in power and status.  10. Patients generally want reassurance rather than information about their health.  15. The patient must always be aware that the doctor is in charge.  17.Humour is a major element of the doctor’s treatment of the patient. |
